# Selective Inguinal Lymphadenectomy in the Treatment of Invasive Squamous Cell Carcinoma of the Vulva

**DOI:** 10.1155/2011/284374

**Published:** 2011-06-09

**Authors:** Christopher P. DeSimone, Jeffrey Elder, John R. van Nagell

**Affiliations:** Division of Gynecologic Oncology, Department of Obstetrics and Gynecology, The University of Kentucky Chandler Medical Center-Markey Cancer Center, 800 Rose Street, Lexington, KY 40536-0293, USA

## Abstract

En bloc radical vulvectomy with bilateral inguinofemoral lymphadenectomy has now been replaced by radical wide excision and selective inguinal lymphadenectomy based on the stage and location of invasive vulvar cancer. Early stage lateral cancers can be effectively treated by radical wide excision and ipsilateral superficial inguinal lymphadenectomy. Lymph node mapping using perilesional injection of radiocolloid and blue dye may identify sentinel lymph nodes which can be removed, thereby avoiding the morbidity of full inguinal lymphadenectomy in selected patients with early stage disease.

## 1. Introduction

Although squamous cell carcinoma of the vulva is not common, it is occurring with increasing frequency in younger women, particularly in those exposed to human papilloma virus (HPV). With efforts at education, many patients are presenting with early stage disease which is amenable to surgery. This year, there will be approximately 3500 new cases of vulvar cancer in the United States, representing 5% of all gynecologic cancers [1]. The surgical management of this disease has evolved from en bloc resection of the entire vulva with bilateral superficial and deep inguinofemoral lymphadenectomy to a more conservative approach involving radical wide excision of the primary lesion and specific types of inguinal lymphadenectomy based on the stage and anatomic location of disease.

## 2. Anatomic Pathways of Spread and Staging

**Figure 1:**
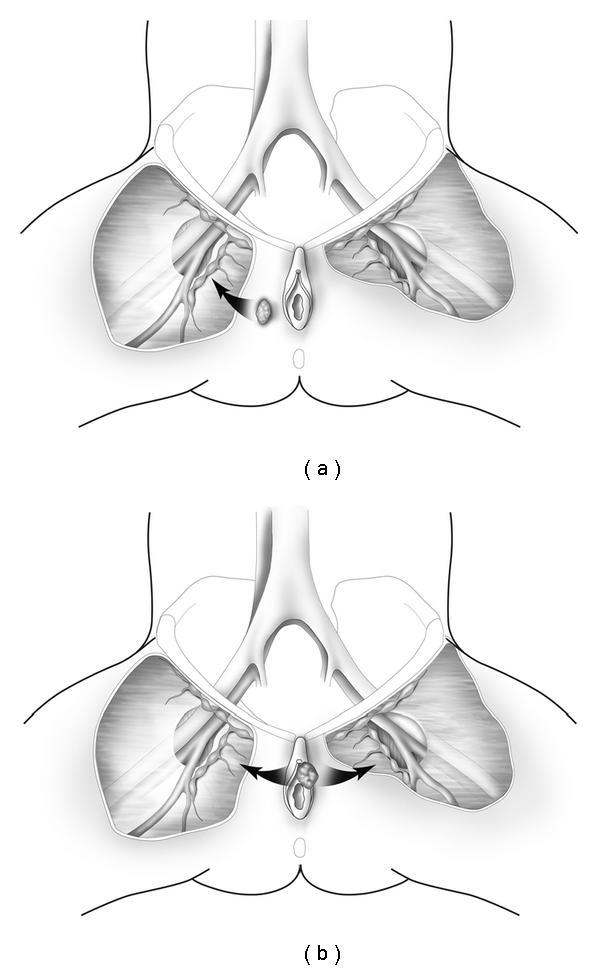
Lateral vulvar lesions >1 cm from the midline (a) spread initially to the ipsilateral superficial inguinal lymph nodes, whereas midline lesions can spread to both groins (b).

The lymphatic drainage of the vulva has been studied extensively and described in numerous publications [2, 3]. Generally, lateralizing lesions (>1 cm beyond the midline) drain to the ipsilateral superficial inguinal lymph nodes, whereas midline lesions can drain to either side (Figure 1). It is extremely rare for lateralizing vulvar cancers to spread to the contralateral inguinal lymph nodes if there is no evidence of ipsilateral lymph node metastases. Likewise, it is unusual for the deep inguinal nodes (below the cribriform fascia) to be involved in the absence of superficial inguinal lymph node spread. The current International Federation of Gynecology and Obstetrics Staging System for vulvar cancer is now based on surgical findings as illustrated in Table 1 and reflects the extent and location of inguinal lymph node metastases. 

## 3. Surgery of the Primary Vulvar Lesion

**Figure 2:**
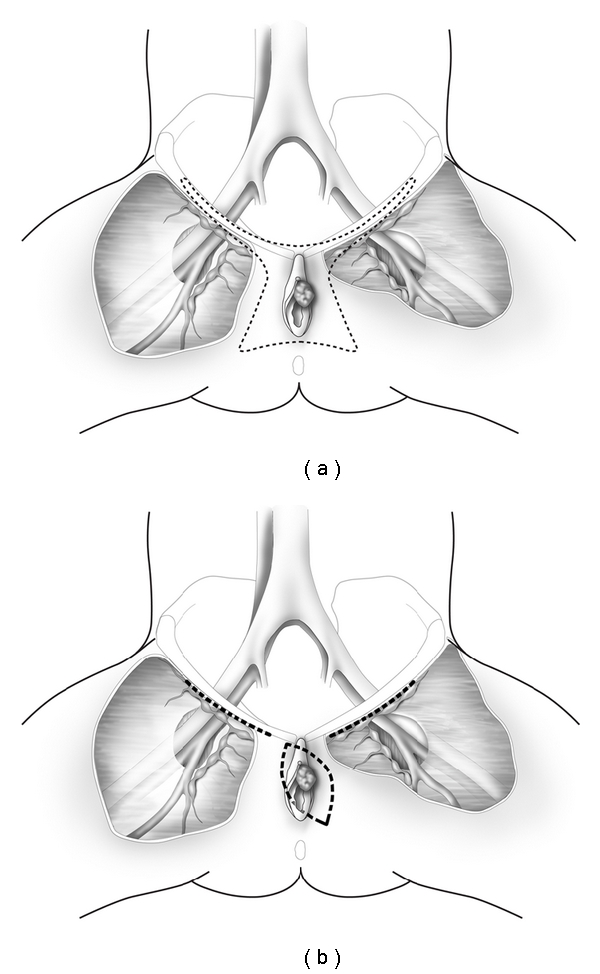
The Bassett-Way operation includes en bloc radical resection of the primary lesion and surrounding vulvar skin as well as the skin over both groins (a). Radical wide excision of the vulvar cancer includes a margin of at least 1 cm of normal skin around the entire lesion. Inguinal lymphadenectomy is performed through separate groin incisions (b).

The Basset-Way operation which was the standard of care in the operative management of patients with vulvar cancer included the en bloc resection of the primary lesion and surrounding vulvar skin as well as the skin over both groins (Figure 2(a)) [4]. Although this procedure was curative in the majority of cases, the wound defect was significant and incisional breakdown and lymphedema of both lower extremities were common. Currently, the surgical treatment of vulvar cancer involves radical wide excision of the primary lesion and inguinal lymphadenectomy through separate groin incisions (Figure 2(b)) [5]. Heaps and colleagues [6] reported that local control of vulvar cancers could be achieved in 100% of cases provided that a 1 cm margin of normal skin was included in the surgical specimen. These observations were confirmed by De Hullu and coworkers [7] who reported that there was no local recurrence of T_1_ or T_2_ vulvar cancers when a margin of at least 8 mm normal skin was excised with the primary lesion. In contrast, patients with a margin of <8 mm had a local recurrence rate of 22%. At present, it is recommended that a margin of at least 1 cm of normal skin around the circumference of the primary vulvar lesion be included in the surgical specimen and that the underlying subcutaneous tissue be removed to the level of the perineal fascia. Inguinal lymphadenectomy is then performed through separate incisions.

## 4. Sentinel Lymphadenectomy

**Figure 3:**
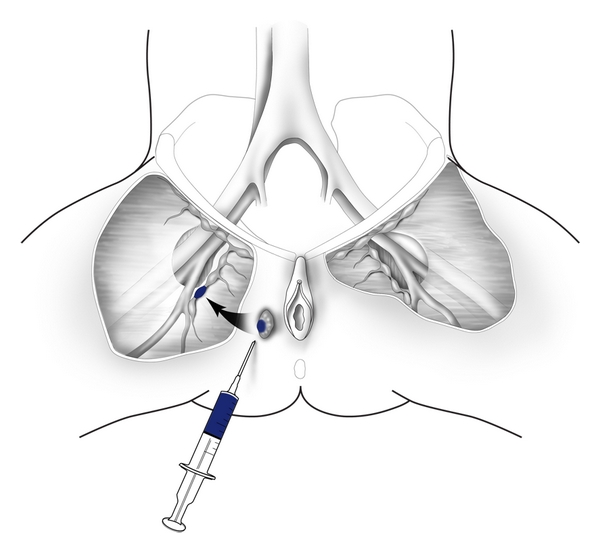
Sentinel lymph node mapping. Sentinel lymph nodes are localized by perilesional injection of 99 mTc radiocolloid and isosulfan blue dye.

Sentinel lymph node excision is now being recommended in selected patients with early stage squamous cell carcinoma as a means to avoid the operative morbidity associated with inguinofemoral lymphadenectomy [8–10]. It is estimated that only 25–30% of patients with early stage vulvar cancer have lymph node metastases, and complete inguinofemoral lymphadenectomy is associated with postoperative wound complications and lower extremity edema in 30–40% of patients [11]. Sentinel lymph node mapping using radiolabelled ultrafiltered sulfur colloid was initially reported as a method to identify regional lymph node metastases in breast cancer [12, 13] and cutaneous melanoma [4] and has now been evaluated in patients with early stage vulvar cancer. Specifically, 1-2 mCi of radiocolloid is injected intradermally around the lesion (Figure 3), and a hand held gamma detection device is used to identify the sentinel lymph node(s). Recently, lymphoscintigraphy has been combined with intraoperative perilesional injection of isosulfan blue as a method to identify sentinel nodes in the inguinal area. Localization of the sentinel node(s) is usually apparent 5–15 minutes after injection of blue dye and 30 minutes after injection of radiocolloid. Selman and colleagues [14] performed a systematic review of the accuracy of sentinel lymph node detection in identifying inguinal lymph node metastases in vulvar cancer and reported that the combination of 99 mTc radiocolloid and isosulfan blue dye successfully detected sentinel lymph nodes in 97% of cases. It also had a negative predictive value (NPV) of 99.1%. Further analysis has indicated that sentinel lymph node mapping is most accurate in patients with early stage lateral vulvar cancers [15]. 

A persisting concern about sentinel lymph node mapping in vulvar cancer is the frequency of groin recurrences in patients with negative sentinel lymph nodes. In a multi-institutional observational study, Van Der Zee and coworkers [10] performed the sentinel node procedure in 623 groins of 403 assessable patients. Two hundred fifty-nine patients with unifocal vulvar cancers <4 cm diameter and negative sentinel nodes were followed without additional therapy for a median of 35 months. Six groin recurrences (2.3%) were observed, and the 3-year survival of these patients was 97%. Importantly, both short-term morbidity and long-term morbidity of patients having sentinel node excision were significantly reduced when compared to that of women undergoing complete inguinofemoral lymphadenectomy. These authors concluded that sentinel node dissection, performed by a quality-controlled multidisciplinary team, should be offered to selected patients with early stage vulvar cancer as a means to avoid the postoperative morbidity associated with inguinofemoral lymphadenectomy.

There is a definite learning curve in the performance and interpretation of lymph node mapping, and it is recommended that a multidisciplinary group within each institution perform sentinel node mapping in at least 10–20 cases before it becomes an accepted procedure [10, 16]. If sentinel lymph nodes cannot be identified by mapping or if there is uncertainty concerning interpretation of findings, a superficial inguinal lymphadenectomy should be performed. 

Patients with a positive sentinel node should undergo a full inguinofemoral lymphadenectomy followed by postoperative radiation therapy to the involved groin and pelvis. However, if the sentinel lymph nodes identified by mapping are histologically negative after review by an experienced multidisciplinary team, no further treatment is indicated. 

It should be emphasized that optimal candidates for sentinel lymph node mapping are patients who have lateral T_1_ or T_2_ unifocal vulvar cancers <4 cm diameter with nonpalpable groin nodes [10]. This procedure is less accurate in patients with midline vulvar lesions or those with advanced stage disease and clinically palpable inguinal nodes [17]. 

## 5. Superficial Inguinal Lymphadenectomy

**Figure 4:**
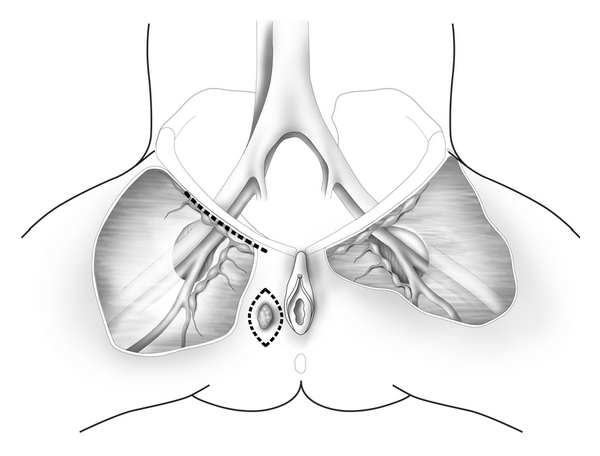
Radical wide excision and ipsilateral superficial inguinal lymphadenectomy is performed for lateral T_1_ or T_2_ vulvar cancers. All lymph nodes above the cribriform fascia are removed and the saphenous vein is preserved.

Many surgeons concerned about the accuracy of sentinel lymph mapping have elected to perform superficial inguinal lymphadenectomy as the treatment of choice in patients with early stage vulvar cancer, believing that the superficial inguinal nodes are themselves “sentinel nodes.” Specifically, all inguinal lymph nodes above the cribriform fascia are removed en bloc (Figure 4). Approximately 8–10 lymph nodes are excised, and the saphenous vein is preserved in order to decrease the frequency of postoperative lower extremity lymphedema [18, 19]. Berman and coworkers [20] reported the outcomes of 50 patients with T_1_ vulvar cancers <1 cm diameter with stromal invasion >5 mm who underwent radical wide excision and superficial inguinal lymphadenectomy. Women with positive superficial inguinal nodes underwent deep inguinal lymphadenectomy and radiation, whereas patients with negative superficial inguinal nodes received no further treatment. There were no isolated groin recurrences noted during a follow-up period of 36 months. Importantly, only 1 patient died of recurrent cancer, and wound complications were observed in only 12% of patients. These authors concluded that radical wide excision of the primary lesion and superficial inguinal lymphadenectomy was the treatment of choice for most women with early stage vulvar cancer and no evidence of enlarged inguinal lymph nodes on clinical examination. A persisting concern of this approach has been a significant but low incidence of groin recurrence in patients with negative superficial inguinal lymph nodes at the time of lymphadenectomy. Two investigators have reported a 4–7% incidence of subsequent ipsilateral groin failure after negative primary superficial groin dissection [21, 22]. This is worrisome since the majority of patients with groin recurrence died of their disease. However, these studies were retrospective and in one series [21] the anatomic location of the primary vulvar lesion was not reported. In the most recent investigation [22], vulvar cancers that recurred in the groin after negative superficial inguinal lymphadenectomy were central periclitoral lesions and the number of lymph nodes removed was small (*∼*3 per groin). Therefore, these authors recommend superficial inguinal lymphadenectomy as the initial surgical approach in patients with lateral Stage I or Stage II vulvar cancers provided that an adequate number of superficial inguinal lymph nodes (8–10) are removed [22]. Currently, superficial inguinal lymphadenectomy should be considered only in patients with lateral T_1_ and T_2_ vulvar cancers having >1 mm stromal invasion and no clinical evidence of enlarged groin nodes.

## 6. Deep Inguinal Lymphadenectomy

**Figure 5:**
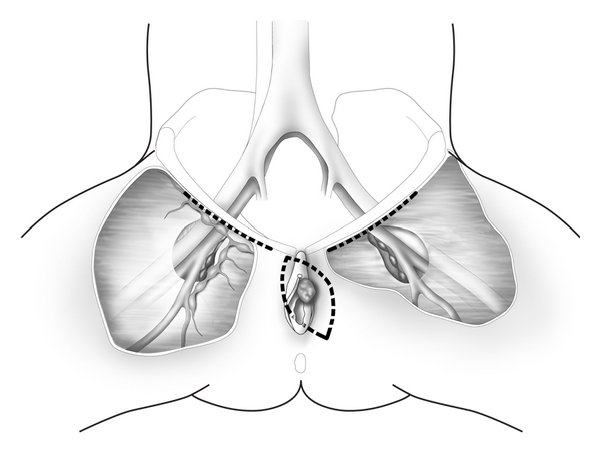
Radical wide excision and bilateral inguinofemoral lymphadenectomy is performed for midline vulvar cancers. This illustration depicts the right superficial inguinal lymph nodes and the left deep femoral lymph nodes which are seen along the medial aspect of the fossa ovalis.

Anatomic studies have indicated that some superficial inguinal nodes may be located within the interstices of the cribriform fascia and could be missed by purely superficial inguinal lymphadenectomy [23]. Therefore, some surgeons choose to perform deep inguinal lymphadenectomy routinely at the time of superficial inguinal lymphadenectomy. The deep femoral lymph nodes are always located within the opening of the fossa ovalis medial to the femoral vein, and no lymph nodes are distal to the lower margin of the fossa ovalis or lateral to the femoral vein (Figure 5). For this reason, incision of the deep fascia of the adductor canal and dissection of the femoral vessels, sometimes performed as part of this procedure, are unnecessary. This is important since deep fascial incision and stripping of the femoral vessels is associated with postoperative wound breakdown, lymphocyst formation, and lower extremity edema in up to 40% of cases [11, 24]. 

At one time, patients with positive superficial or deep inguinal nodes were treated by pelvic lymphadenectomy. However, a prospective trial conducted by the Gynecologic Oncology Group showed that patients with inguinal lymph node metastasis at the time of groin dissection who were treated by postoperative inguinal and pelvic radiation had a significant survival advantage when compared to similar patients treated by pelvic lymphadenectomy [25]. Patients in the radiation therapy arm received 45–50 Gy to the involved groin and pelvis, whereas patients randomized to pelvic lymphadenectomy underwent a standard extraperitoneal excision of the obturator, external iliac, internal iliac, and common iliac lymph nodes. This trial showed a statistically significant survival advantage to patients in the radiation therapy arm (68% versus 54% observed survival) on interim analysis [26]. Therefore, most vulvar cancer patients with positive superficial or deep inguinal lymph node metastases at the time of inguinal lymphadenectomy are treated now by postoperative radiation to the involved groin and pelvis. 

## 7. Unilateral versus Bilateral Inguinal Lymphadenectomy

Anatomic studies have confirmed that efferent lymphatics from the lateral vulva drain initially to the ipsilateral superficial inguinal lymph nodes [2, 27]. Likewise, lateral vulvar cancers (>1 cm beyond the midline) do not spread to the contralateral inguinal lymph nodes without first involving the ipsilateral inguinal nodes [27, 28]. These anatomic observations have led to clinical trials evaluating the efficacy of radical wide excision and ipsilateral inguinal lymphadenectomy in the treatment of lateral vulvar cancers. DeSimone and coworkers [29], for example, treated 122 patients with lateral T_1_ or T_2_ vulvar cancers by radical vulvectomy (*N* = 60) or radical wide excision (*N* = 62) and bilateral inguinal lymphadenectomy. Twenty-six patients (21%) had ipsilateral inguinal lymph node metastases, but there were no cases of spread to the contralateral inguinal lymph nodes. Patients with positive inguinal nodes were treated by postoperative radiation therapy to the involved groin and pelvis. All patients in the study were followed periodically by clinical examinations 10–195 months (mean 59 months) after treatment. Eighteen patients (15%) developed recurrent vulvar cancer—13 to the ipsilateral vulvar skin, 2 to the ipsilateral groin, and 3 to the lung. There were no recurrences to the contralateral vulvar skin or groin. Likewise, there was no difference in the local recurrence rate of patients treated by radical vulvectomy versus those treated by radical wide excision. The 5-year disease-free survival was 98% for patients with T_1_ lesions and 93% for patients with T_2_ lesions. These authors concluded that women with lateral T_1_ and T_2_ vulvar cancers could be treated effectively by radical wide excision and ipsilateral inguinal lymphadenectomy.

There is still some controversy concerning the performance of routine contralateral inguinofemoral lymphadenectomy in patients with lateral vulvar cancers and positive ipsilateral inguinal nodes. Isolated reports have indicated that contralateral inguinal lymph node metastases can occur in these patients. However, the frequency of contralateral spread in these patients is extremely low, and the risk of contralateral inguinal lymphadenectomy may outweigh its benefits, particularly if only a small number of ipsilateral nodes are positive. 

All vulvar cancers located within 1 cm of midline structures (clitoris, vagina, or anus) have the potential to spread to both groins, and should be treated by radical wide excision and bilateral inguinofemoral lymphadenectomy. 

## 8. Summary

The Bassett-Way operation, which emphasized en bloc resection of the vulva and both groins, has been replaced by radical wide excision and selective inguinal lymphadenectomy through separate groin incisions. The specific type of inguinal lymphadenectomy indicated depends on the stage and location of each cancer. Early stage lateral vulvar cancers can be treated safely by radical wide excision and ipsilateral superficial inguinal lymphadenectomy, whereas central vulvar cancers require bilateral inguinal lymphadenectomy. Deep inguinal lymphadenectomy, which involves surgical removal of the deep femoral nodes, is indicated in patients with centrally located early stage vulvar cancers, advanced stage vulvar cancers, and in patients with positive superficial inguinal lymph nodes. The deep femoral lymph nodes are always located medial to the femoral vein and can be removed without incising the deep fascia or dissecting the femoral vessels. Sentinel lymph node mapping should be offered to select patients with early stage lateral vulvar cancers as a means to avoid the postoperative morbidity associated with full inguinofemoral lymphadenectomy. 

## Figures and Tables

**Table 1:** FIGO staging of invasive cancer of the vulva.

Stage I	Tumor confined to the vulva
IA	Lesions ≤2 cm in size confined to the vulva or perineum and with stromal invasion ≤1.0 mm*, no nodal metastasis
IB	Lesions >2 cm in size confined to the vulva or perineum with stromal invasion greater than 1.0 mm*, no nodal metastasis

Stage II	Tumor of any size with extension to adjacent perineal structures (1/3 lower urethra, 1/3 lower vagina, anus), no nodal metastasis

Stage III	Tumor of any size with or without extension to adjacent perineal structures (1/3 lower urethra, 1/3 lower vagina, anus) with positive inguinofemoral lymph nodes
IIIA	With 1 lymph node metastasis (≥5 mm),
IIIB	(i) With 2 or more lymph node metastases (≥5 mm), (ii) 3 or more lymph node metastases (<5 mm)
IIIC	With positive nodes with extracapsular spread

Stage IV	Tumor invades other regional (2/3 upper urethra, 2/3 upper vagina) or distant structures
IVA	Tumor invades any of the following: (i) Upper urethra and/or vaginal mucosa, bladder mucosa, rectal mucosa, or fixed to pelvic bone, (ii) Fixed or ulcerated inguinofemoral lymph nodes
IVB	Any distant metastasis including pelvic lymph nodes

*The depth of invasion is defined as the measurement of the tumor from the epithelial-stromal junction of the adjacent most superficial dermal papilla to the deepest point of invasion.
